# Cluster Analysis of Residential Personal Exposure to ELF Magnetic Field in Children: Effect of Environmental Variables

**DOI:** 10.3390/ijerph16224363

**Published:** 2019-11-08

**Authors:** Gabriella Tognola, Emma Chiaramello, Marta Bonato, Isabelle Magne, Martine Souques, Serena Fiocchi, Marta Parazzini, Paolo Ravazzani

**Affiliations:** 1 CNR IEIIT—Consiglio Nazionale delle Ricerche, Istituto di Elettronica e di Ingegneria dell’Informazione e delle Telecomunicazioni, 20133 Milan, Italy; emma.chiaramello@ieiit.cnr.it (E.C.); marta.bonato@ieiit.cnr.it (M.B.); serena.fiocchi@ieiit.cnr.it (S.F.); marta.parazzini@ieiit.cnr.it (M.P.); paolo.ravazzani@ieiit.cnr.it (P.R.); 2 EDF Electricité de France, 75017 Paris, France; isabelle.magne@edf.fr (I.M.); martine.souques@edf.fr (M.S.); 3 Dipartimento di Elettronica, Informazione e Bioingegneria DEIB, Politecnico di Milano, 20133 Milan, Italy

**Keywords:** children, ELF MF, magnetic field, residential exposure, cluster analysis, machine learning, electric heating, residence age, residence type, family size

## Abstract

Personal exposure to Extremely Low Frequency Magnetic Fields (ELF MF) in children is a very timely topic. We applied cluster analysis to 24 h indoor personal exposures of 884 children in France to identify possible common patterns of exposures. We investigated how electric networks near child home and other variables potentially affecting residential exposure, such as indoor sources of ELF MF, the age and type of the residence and family size, characterized the magnetic field exposure patterns. We identified three indoor personal exposure patterns: children living near overhead lines of high (63–150 kV), extra-high (225 kV) and ultra-high voltage (400 kV) were characterized by the highest exposures; children living near underground networks of low (400 V) and mid voltage (20 kV) and substations (20 kV/400 V) were characterized by mid exposures; children living far from electric networks had the lowest level of exposure. The harmonic component was not relevant in discriminating the exposure patterns, unlike the 50 Hz or broadband (40–800 Hz) component. Children using electric heating appliances, or living in big buildings or in larger families had generally a higher level of personal indoor exposure. Instead, the age of the residence was not relevant in differentiating the exposure patterns.

## 1. Introduction

Personal exposure to Extremely Low Frequency Magnetic Fields (ELF MF, 50/60 Hz) in children has raised particular interest since the late 80s [1] when ELF MF exposure was found to be a potential risk factor for childhood leukemia. In the first decade of the 2000s, some studies [2,3,4] showed that the risk of childhood leukemia increased for daily average MF exposures >0.4 μT, without evidencing a causal relationship. Since then, an ever increasing number of studies and campaigns were implemented throughout the world to measure ELF MF in children and thus provide realistic data about the level of personal exposures in everyday settings [5,6,7,8,9,10,11,12,13,14,15,16,17,18].

Of particular interest is to understand which variables contribute mostly to personal exposure to ELF MF. Aim of the present study is to analyze how indoor personal exposure in children could be affected by environment variables including not only electric networks in close proximity to the child home but also other ‘secondary’ variables that might potentially influence indoor personal exposure, such as the use of electric heating, the residence type (individual vs. apartment houses), the residence age, and the family size.

Differently from the majority of previous studies on the analysis of ELF MF exposure [5,6,7,8,9,10,11,12,13,14,15,16,17,18], we did not perform a parametric modeling by using, e.g., correlation analysis or multivariate analysis but we applied cluster analysis—a machine learning technique—to identify recurrent and common patterns of indoor personal exposures. Cluster analysis is a non-parametric approach which is used to perform exploratory analysis to discover possible hidden patterns in the observed data. Differently from parametric analyses such as the well-known correlation, regression, and factor analysis, cluster analysis does not require that the data come from a parametric model (linear or non-linear): cluster analysis aims at partitioning the observed data in groups so that members of a given group share more similarities among them if compared with the members of the other groups. It is a consolidated practice to use cluster analysis to complement parametric methods to discover additional and useful information.

The present study is the follow-up of our previous work [19] in which machine learning was applied for the first time in the field of ELF MF exposure assessment. In the present study we wanted to answer to a number of open questions that we did not address in our past preliminary study. Namely: (i) we aimed to understanding how the patterns of indoor personal exposure found by cluster analysis were affected by the presence of indoor sources of ELF MF such as electric heating and by variables that might indirectly contribute to indoor exposure such as the physical parameters of the residences (the age and type of residence (i.e., individual vs apartment )) and the family size; and (ii) we analyzed not only the 50 Hz MF (as in [19]) but also the broadband (40–800 Hz) and harmonic (100–800 Hz) components to assess their potential contribution in differentiating the patterns of indoor personal exposure. Most of the previous studies on personal exposure to MF have only measured the broadband component (and the 50 Hz component, which was calculated from the broadband signal). With the exception of high voltage power lines where the MF is purely 50 Hz, most of ELF magnetic field sources have a part of 50 Hz and a part of harmonics. The objective of this study was to improve the characterization of the exposure to ELF MF by analyzing both broadband and harmonic components. Also, the presence of many electronic devices (TV receivers, computers, domestic electrical devices) creates a high level of harmonic content mainly because of their switching power supplies. Measuring the harmonic component is thus potentially useful to identify MF sources other than electric networks. Finally, in the present study we also considered the possible effect of uncertainty in geolocalization of electric networks near children homes.

It is worthwhile to note that, due to the use of cluster analysis, the results that will be described in the paper are exploratory; as such they cannot test hypotheses but can only identify hypotheses.

## 2. Materials and Methods

### 2.1. Dataset Description

The analyzed dataset consisted of 24h indoor measurements of ELF MF (magnetic flux density B) personal exposure in children living in France. Exposure was measured with a personal exposimeter (EMDEX II, Enertech, Campbell, CA, USA; sensitivity: 0.01–300 μT; sampling rate 3 s). The measurement device was set up to measure the broadband, harmonic, and the fundamental (i.e., 50 Hz) component of the MF. As explained in the technical documentation of the exposimeter, the fundamental *Fc* is calculated from the broadband and harmonic components as follows:

(1) $$\begin{array}{cc} Fc=\sqrt{B_{m}^{2}-H_{m}^{2}} & H_{m}<B_{m}\\ Fc=0 & H_{m}\geq B_{m} \end{array}$$

where $B_{m}$ and $H_{m}$ are the RMS values of the broadband and harmonic components measured by the exposimeter.

As in [19], the set of data come from the EXPERS study [5,6]; the detailed protocol of the EXPERS study can be found in [5]. Personal exposure was measured during 24 h every 3 s; subjects were asked to worn the EMDEX meter during 24 h, except during night where the meter was to be put close to the bed. The dataset contained one recording per child and only one child per home. The EXPERS dataset contained the number of electric lines close to the child position for the following type of networks: 400 V underground cables at a distance ≤40 m (UNDlow); 20 kV underground cables at a distance ≤40 m (UNDmid); 63–150 kV underground cables at a distance ≤20 m (UNDhigh); 225 kV underground cables at a distance ≤20 m (UNDextra); 400 V overhead lines at a distance ≤40 m (OVHDlow); 20 kV overhead lines at a distance ≤40 m (OVHDmid); 150 or 90 or 63 kV overhead lines at a distance ≤100 m (for 150 kV lines) or 70 m (for 63 and 90 kV lines) (OVHDhigh); 225 kV overhead lines at a distance ≤120 m (OVHDextra); 400 kV overhead lines, at a distance ≤200 m (OVHDultra); 20 kV/400 V substations inside the building at a distance ≤40 m (Substation).

The present analysis was performed on the recordings of 884 out of 979 of the children available in dataset, because for this subset of children the database reported the type of heating used at home, the age and type of the home, and the family size. For these 884 children, we extracted and analyzed those portions of the original 24 h recording that were measured when the child was at home.

The measurement device does not have GPS; thus the location of the device was inferred from the information provided by the subjects, who were asked to specify the periods during the 24 h when they were at home or at school. To exclude any bias due to the presence of the alarm clock in exposure measurements done at night (as reported in [6]), we extracted and analyzed from each recording only the period measured during the day, as identified in the activity report provided by each child. On average the length of this period was 6 hours. The analyzed dataset also reports the precision of the geolocalization of the child home. The precision was the highest when the exact address (and civic number) of the home was known. The lowest precision corresponded for addresses which were not defined by a precise address but were localized in a so-called “commune”, which is the lowest administrative division in France (there are around 36 000 communes in France, each one has a mayor). The precision in the geolocalization of the child home in our dataset ranged from 15 m (highest accuracy) to 1000 m (lowest accuracy). For what concerns electric networks, geolocalization was provided by the French grid operators (RTE for high voltage and Enedis for mid and low voltage) and calculated from the Lambert II coordinates of home address. For RTE network, the coordinates of the pylons are identified with a geographical uncertainty ranging from 2.5 to 25 m. At the present moment, 90% of RTE pylons are localized with an uncertainty of 2.5 m [20]. The precision for Enedis network was lower than for RTE and around 20 m.

### 2.2. Data Analysis: Clustering and Association Effect Analysis

We first performed a preliminary analysis of our dataset to assess how robust would be performing the clustering using all the environmental variables at a time. To evaluate the robustness, we used two parameters, namely the correlation ratio *η*^2^ [21] and the silhouette score [22]. The correlation ratio *η*^2^ evaluates the importance of a given variable in differentiating the clusters; it is calculated as the ratio between the variance of the variable explained by cluster membership and the variance across the whole population sample:

(2) η2=∑cnc(x¯c−x¯)2∑i(xi−x¯)2

where ${\bar{x}}_{c}$ is the mean value of the variable calculated within cluster *c* (c=1,⋯,C), $\bar{x}$ is the mean over the whole population $x_{i}$ (i=1,⋯,884), $n_{c}$ is the size of cluster *c*, and *C* is the number of clusters. The correlation ratio ranges from 0 to 1; the higher the greater the contribution of that variable in discriminating the clusters. The silhouette score is a measure of the overall appropriateness of the cluster solution: the higher the more appropriate is the solution. The preliminary analysis (results are not reported in the present paper) revealed that our variables were not equally important in differentiating the clusters: as a matter of fact there was a subset of variables (those we called ‘primary’) that clearly had a correlation ratio much greater than others (the ‘secondary’ variables). These latter variables were characterized by very low correlation ratios. Besides this, we observed that the clustering obtained by considering all the variables at a time was poorer when compared to the that one obtained by using only the primary variables: as a matter of fact the silhouette score obtained when using all the variables was lower than when using only the subset of the primary variables. These two findings together (i.e., lower correlation ratio + lower silhouette score) were a clear indication that it would be more appropriate in our study to use a two-step approach as described below.

In the two-step approach, first we applied cluster analysis on the variables that were found to be ‘primarily’ responsible of the generation of the clusters and second we applied an association effect analysis to further and deeper investigate the potential (and secondary) effect of the secondary variables on the clusters already found in step one. The results of the preliminary analysis strongly suggest that it would not be robust to perform the cluster analysis on the whole set of variables at once, i.e., primary + secondary, because this would lead to erratic clustering results that would be affected by the lower strength of the secondary variables in generating the clusters.

The set of ‘primary’ variables (as identified during the preliminary analysis) included the number of electric networks close to the child location (i.e., variables from ‘UNDlow’ to ‘substation’) and the geometric mean (GM) of B measured indoor calculated as:

(3) $$\mathrm{GM}=\exp\left(\frac{1}{N}\sum_{i=1}^{N}\log(B_{i})\right)$$

where B_*i*_ is the value of the magnetic flux of the *i*-th sample (i=1,⋯,N). In (3), we replaced the null values of B*i* by 0.00625 µT (which corresponds to half the resolution of the EMDEX II). The set of ‘secondary’ variables, included house heating, residence age, residence type, and family size.

To perform cluster analysis, we followed the same procedure already used in [19]. Data were clustered with the *K*-means algorithm (Matlab, ver. R2018a, MathWorks Inc., Natick, MA, USA) [22,23]. The *K-*means algorithm subdivides the observations in mutually exclusive and exhaustive groups —the clusters—so that observations within the same group are more similar to each other than those in the other groups (for more details on the mathematics behind this approach, see [19]). The similarity between observations was measured by the squared Euclidean distance. In *K*-means, each cluster is characterized by a center point—the centroid—which is the ‘representative’ of the characteristics of the observations belonging to that cluster and is calculated as the mean of the observations assigned to that cluster. We generated three different cluster models, one for the 50 Hz, another for the broadband (40–800 Hz) and the last one for the harmonic (100–800 Hz) component of B. Similarly to [19], *K* (the optimal number of clusters) was determined by choosing among all the possible cluster solutions that one that maximized the silhouette score and led to reasonable clusters’ size. In this work, we accepted those solutions characterized by a cluster size of at least 5 observations in each cluster. Application of the above rule to our dataset led to an optimal solution with *K* = 3, which was characterized by an average silhouette of 0.60 (indicative of a cluster solution of good quality) and had at least seven observations per cluster.

To assess the potential effect of secondary environmental variables on the clusters, we applied the Chi-square (*χ*^2^) association test on the categorical variables (house heating, residence age, and residence type) and the Kruskal-Wallis test on the numerical variable (family size). The *χ*^2^ test assesses the null hypothesis (H_0_) that the distribution of a categorical variable discretized in *R* categories is equally distributed over the *K* clusters; the significance level to reject H_0_ was set at *p* < 0.05. Roughly speaking, if, for a given variable, H_0_ can be rejected this means that the distribution of that variable depends on the clusters, i.e., there is a statistically significant association between that variable and the clusters. The variable “heating” was discretized into *R* = 3 categories, namely: non-electric, mixed, and electric heating. The variable “residence type” was discretized into *R* = 4 categories, namely: individual, terraced, apartment in small building (with two to nine residential units), and apartment in big building (with 10 or more residential units). Finally, the variable “residence age” was discretized into *R* = 4 categories based on the building year, namely: before 1950, from 1950 to 1969, from 1970 to 1989, and after 1989.

For those variables for which H_0_ was rejected, we measured the degree of association between the variable and the clusters with the Effect Size (ES) index *w* [24]:

(4) $$w=\sqrt{\overset{R}{\underset{r=1}{\sum }}\overset{K}{\underset{k=1}{\sum }}\frac{{\left(P1_{r,k}-P0_{r,k}\right)}^{2}}{P0_{r,k}}},$$

where *P*1*_r,k_* is the proportion of data in category *r* (r=1,⋯,R) really assigned to cluster *k* (k=1,⋯,K) and *P*0*_r,k_* is proportion of data in category *r* that would be expected in cluster *k* if H_0_ were true (i.e, if the distribution did depend on the cluster). *w* ranges from 0 to $\sqrt{\min\left(R-1,K-1\right)};$ the higher, the greater the association of that variable with the clusters. Typically, w≤0.10 is regarded as a small effect, w=0.30 as a medium and w≥0.50 as a large effect. As *w* depends on the size of *R* and *K*, it is common practice to complement the analysis with generalized and normalized measures of ES. In the present study we used the Cramer’s *V* [24]:

(5) $$V=\frac{w}{\sqrt{\min\left(R-1,K-1\right)}},$$

where *V* ranges from 0 to 1.

Finally, we applied the Kruskal-Wallis test to assess the null hypothesis (H_0_) that family size (i.e., the number of household residents) over the *K* clusters was the same; the test was considered statistically significant at *p* < 0.05. In case that H_0_ was rejected, we applied the post-hoc Dunn’s test for performing multiple pairwise comparisons among the *K* clusters.

## 3. Results

The median GM over the whole dataset of 884 children was 0.010 μT @50 Hz (1st quartile Q1: 0.003 μT; 3rd quartile Q3: 0.028 μT), 0.012 μT @broadband component (Q1: 0.004 μT; Q3: 0.033 μT), and 0.002 μT@ harmonic component (Q1: 0.001 μT; Q3: 0.005 μT). GM was >0.4 μT only in four (4) recordings out of 884. As illustrated in Table 1, most children in our sample (72.4%) live near underground cables of low voltage, about 50% live near overhead lines of low voltage, and nearly 46% are near underground cables of mid voltage. About 14% of the children live near substations and 4.5% are near overhead lines of mid voltage. Less than 1% lived near underground cables of high and extra-high voltage or overhead lines of high to ultra-high voltage. For low and mid voltage lines, the number of networks is more exactly the number of portion of cables, as described in the Enedis geographic information system. This number can be high in areas with a lot of interconnections, like in Paris.

Table 2, Table 3 and Table 4 display the three models obtained by clustering the 50 Hz, broadband, and harmonic components of the indoor MF separately. Each model was obtained by partitioning the data into three clusters because, as previously described in the Methods, the optimal number of clusters was found to be *K* = 3.

The three models displayed in Table 2, Table 3 and Table 4 have several similarities, especially regarding the characteristics of the clusters. As a matter of fact, all the three models show that: Cluster 1 had the smallest size and grouped together children with the highest residential exposures and the highest number of 63 to 400 kV overhead lines close to their home; Cluster 2 had a medium size and consisted of children with the highest number of substations and underground networks of low and mid voltage near home. Finally, cluster 3 had the largest size and consisted of children with the lowest number of electric networks near home. Cluster 3 also contained the four cases with GM >0.4 μT. When looking more into details these four cases, the MF source explaining the high values of residential exposure was for one child the presence of low voltage overhead line with bare and spaced conductors close to the home, for another child the presence of 25 kV 50 Hz train line close to home, and for the remaining two children the presence of electric devices (for one of them it could be an aquarium and for the other child it could be a small transformer) close to the exposimeter. This illustrates the fact that the clusters are an averaged description of the characteristics of the children in each cluster: this means that Cluster 3 is not simply the cluster with the lowest exposures or with no electric network around the home.

The average GM in the three clusters of the three models displayed in in Table 2, Table 3 and Table 4 are different, with higher average GM values observed in the model of the broadband component (i.e., Table 3): the average GM in Cluster 1 (highest residential exposures) was 0.126 μT @50 Hz, 0.129 μT @broadband component, and 0.010 μT @harmonic component; the average GM value in Cluster 2 (mid residential exposure) was 0.036 μT @50 Hz component; 0.041 μT @broadband component; 0.007 μT @harmonic component; and, finally, the average GM in Custer 3 (lowest residential exposure) was 0.025 μT @50 Hz component; 0.028 μT @broadband component; 0.004 μT @harmonic component.

The last column in Table 2–4 displays the correlation ratio *η*^2^ which, as explained in the Methods, is a parameter that measures how the variables are important in differentiating the three clusters: the higher *η*^2^ the higher the contribution of that variable in differentiating the cluster. It is seen that underground networks of low and mid voltage, overhead lines of high to ultra-high voltage, and substations have higher *η*^2^ than underground networks of high and extra-high voltage and overhead lines of low- to mid-voltage, thus meaning that these former variables have a greater contribution in differentiating the clusters than the latter ones. As to the contribution of GM, the broadband and 50 Hz components contributed more than the harmonic component to the clusters.

The following paragraphs describe the results obtained from the analysis of the secondary environmental variables. In particular, Table 5 shows the results of the *χ*^2^ test which evaluated the potential association between the secondary environmental variables (i.e., heating, residence type, and residence age) and the clusters. It can be seen that heating and the type of residence had a statistically significant association with the clusters (*p* < 0.005), whereas the residence age has no association. Moreover, as evidenced by *w* and *V* (see again Table 5), the residence type had a stronger association with the clusters than heating.

Table 6, Table 7 show the detailed analysis of the joint distributions of heating and residence type (i.e., of those variables that were found to have a statistically significant association with the clusters) across the clusters. This analysis is useful to understand how the different types of heating and residences are distributed across the clusters. Results displayed in Table 6 confirmed that heating type is not equally distributed across the clusters (this was expected because the *χ*^2^ test on cluster association (see previous Table 5) revealed that heating was associated to the clusters): as a matter of fact it can be observed from Table 6 that there were more cases than expected with electric heating in Cluster 2 (4.40% instead of 3.23%, corresponding to 38 children instead of 28) and more cases then expected with mixed heating in Cluster 3 (16.30% instead of 14.55%, corresponding to 141 children instead of 126). This means that electric heating was mostly associated to Cluster 2 (mid residential exposures), whereas mixed heating was mostly associated to Cluster 3 (lower residential exposures). It is to note that with ‘electric heating’ we intended the use of electric appliances to heat the home, such as electric radiators, and not only the use of floor heating.

Similarly to heating, the residence type was differently distributed across the clusters. As a matter of fact, the results displayed in Table 7 show that the distribution of the number of children living in individual and terraced houses in Cluster 3 was greater than expected, whereas those living in big buildings were greater than expected in Cluster 2; this means that individual and terraced houses were mostly associated to children assigned to Cluster 3 (lowest residential exposures), whereas big buildings were mostly associated to children in Cluster 2 (mid residential exposures).

A closer inspection of the data revealed that there was a statistically significant association between the place of residence and the clusters (*p* < 0.001; *η*^2^ = 72.62; *w* = 0.29; *V* = 0.20): as a matter of fact, there were more cases than expected living in remote areas in Cluster 3 and, vice versa, more cases than expected living in big (>2 millions of inhabitants) urban units in Cluster 2. If we put together all these evidences, we can state that Cluster 2 is characterized by children with mid-level exposures, who live in big cities and big buildings located near underground electric networks and substations.

Family size was found to differ with the clusters (*p* < 0.02; *η*^2^ = 8.63); in particular, family size was greater for children assigned to Cluster 2 than in Cluster 3 (Dunn’s post-hoc test, *p* < 0.005), whereas no statistically significant differences were found between Cluster 1 and Cluster 2 or 3.

The analysis of the dataset revealed that the precision in home geolocalization was 15 m for the majority of the data (75% of 884 measurements), 50 m in 5.20% cases, 300 m in 10.75% cases, 500 m in 8.26% cases, and 100 m or 1000 m for less than 0.5% cases, only. Precision was about 20 m for low and middle voltage networks, and about 15 m for high to ultra-high voltage networks. The *χ*^2^ test revealed that precision differed across the clusters (*p* < 0.04); however, the ES was small (*w* = 0.15, *V* = 0.10). By applying the analysis of the joint distribution of precision across the clusters (as we did Table 6 and Table 7), we found that only Cluster 2 and 3 had slightly lower precisions than expected: in Cluster 2 there were 0.11% cases with a precision of 1000 m instead of 0.05% as expected if precision had distributed uniformly across the clusters, whereas in Cluster 3 the cases with a precision of 500 m were 8.14% instead of 6.83%. We can conclude that, although statistically different, the degree of precision can be considered almost the same over the clusters, with no practical effect.

## 4. Discussion

Cluster analysis revealed that the number and type of electric networks near child home contributed to characterize indoor personal exposure. The first pattern (Cluster 1) was characterized by children with the highest indoor exposures, living near overhead lines of high (63–150 kV), extra-high (225 kV) and ultra-high voltage (400 kV). The second pattern (Cluster 2) was related to children with mid exposure levels, living near underground networks of low (400 V) and mid voltage (20 kV) and substations (20 kV/400 V). It is to note that in France, underground networks are mainly found inside the cities, as illustrated in [6]. Finally, the third pattern (Cluster 3) characterized children with the lowest exposure living more distant from electric networks. Similar patterns were observed also in our previous study [19] on a larger sample of 1794 personal measurements of indoor ELF exposures made at child’s home and in schools; differently from the present study, in [19] we identified two different clusters for children with high exposures: one for children near extra-high and ultra-high voltage lines who were characterized by very high exposures and another for those living near high voltage lines who were characterized by mid-to-high exposures. The reason of this slight different clustering between the present and the former study could be due to the lower number of children living near high to ultra-high electric lines in present dataset (14 cases instead of 23 because in the present study we did not consider exposure measurements done at schools) which might have made less ‘convenient’ for the *K*-means algorithm splitting these cases into two separate clusters. It is to note that Cluster 2 was characterized by children living near underground network of low to mid voltage and substations: this combination of underground network and substations is not bizarre because according to the typical configuration of electric grids, substations are typically found close to underground networks of low and mid voltage.

The present analysis revealed that the harmonic component of B was not relevant alone in differentiating the three patterns of indoor exposure. As a matter of fact, harmonics are negligible for 63 to 400 kV lines and often (but not always) for low and mid voltage line. Vice versa, they are strictly linked to the magnetic field generated by small electric appliances used in each home (and can be viewed as signatures of the specific spectrum emitted by a given electric appliance). As such, although harmonics could not be used alone to differentiate the exposure patterns identified in the three clusters, they nonetheless could be useful in characterizing more precisely the sources of indoor MF other than electric networks that contributed to a lesser extent to the overall exposure.

As revealed by the correlation ratio *η*^2^ (see Table 2, Table 3 and Table 4), underground networks of low to mid voltage, overhead lines of high to ultra-high voltage, and substations contributed in differentiating the exposure patterns, whereas underground network of high (63–150 kV) and extra high voltage (225 kV) and overhead lines of low (400 V) to mid voltage (20 kV) have only a marginal discrimination power. This result is in line with what we observed in [19].

We found that electric heating was mainly associated to Cluster 2, i.e., to mid-levels of indoor MF exposure. Vice versa, mixed heating was associated to the lowest exposure levels. It is to note that with electric heating we intended in our analysis not only the use of electric floor heating but also other appliances, such as electric radiators. This association of some types of electric heating with higher indoor ELF exposure was also evidenced in previous studies: as reviewed in [25], a survey commissioned by the Swiss Federal Office of Public Health in 2016 [26] reported that the MF produced indoor by electric floor heating was well below the threshold of 100 μT, although it was higher than that normally measured in residential settings in Switzerland [27]. Similarly, in an older study conducted by the Electric Power Research Institute (EPRI) [28], electric heating in the ceilings or floors was identified as a source of the MF measured indoor in about 1000 residences in US. In particular, electric heating in the ceilings produced a MF greater than that of most other ELF MF sources in the room and ranged from 2.6 μT (median value) at 30 cm from the ceiling to 0.3 μT near the floor.

We found no association between the residence age and indoor personal exposure. Past surveys led to inconsistent evidences on this matter. For example, in the US survey by EPRI [28], higher MFs were found in older residences as a result of the greater prevalence of particular ‘old fashioned’ wiring solutions, such as knob-and-tube wiring, that can produce considerably higher fields. Vice versa, in a survey conducted in Spain [29], residential daytime MFs were significantly higher in new houses than in older ones. The authors of this latter study did not provide any comment to explain this result. The year of construction of the home was found to be correlated to the magnetic field indoor, as the proximity to 63 to 400 kV overhead line, in the department Côte d’Or in France [30]. However this department is mainly rural and not representative of the whole France.

Our results revealed that the residence type was associated to the clusters. In particular, we found a greater prevalence of individual and terraced residences in the cluster with lower exposure levels (Cluster 3) and residences in big buildings in the cluster with mid-exposure levels (Cluster 2). Further analysis of our dataset revealed that the association between the residence type and the clusters was not entirely a direct consequence of the level of MF exposure rather a consequence of parameters that are related to both housing density and MF exposure. As a matter of fact, we found that children living in big buildings generally lived in high urbanized areas and that the most frequent type of networks in such areas were underground lines. As illustrated before, underground networks characterized Cluster 2, i.e., that one with mid exposure levels. Vice versa, we found that children living in individual or terraced houses typically live in less urbanized centers and that their homes were more distant from electric networks. These children were characterized by low MF exposures and were thus assigned to Cluster 3. Similar results were described in previous studies (e.g., [28,31,32]), where lower MF were generally found in single-family houses.

Our analysis identified a statistically significant association between the size of the family and personal MF exposure: children with mid-level exposures (Cluster 2) were living in larger families than those with lower exposures (Cluster 3). Cluster 3 is more in single house so more in rural areas. There is a movement of population in France leaving rural areas to go to big cities. So in rural areas the population is older and with less children. We found no statistically significant difference of the family size between children with the highest exposures in Cluster 1 and those ones in the other clusters.

As illustrated in the Results, we found from the association effect analysis that the uncertainty due to geolocalization could be considered the same over the clusters. This, from a statistically point of view, would mean that we can expect that the impact of uncertainty in the geolocalization would be almost the same across the clusters. From a practical point of view, however, we cannot exactly quantify how much is this error and how much it might impact the clusters. We know that in our dataset measurements were done with a geolocalization accuracy within 15 m in 75% of the cases and greater than 15 for the remaining 25%. This means that the majority of the networks in our dataset were localized with the highest accuracy. However, because of the uncertainty in the geolocalization, the likelihood that a voltage line might have been mis-classified is not null, especially for those lines within 20 m from child home, i.e., UNDhigh and UNDextra. The dataset contained eight children who lived close to UNDhigh and/or UNDextra networks: for seven children, networks were localized with a precision of 15 m (i.e., with the most accurate level); for the last child the precision was 50 m. So, we can say that, for what concerns the precision in the geolocalization of UNDhigh and UNDextra networks, seven out of eight measurements in our dataset come from locations with the highest accuracy. From the analyses presented in the current study, we found that UNDhigh and UNDextra networks have less influence on our clustering results than OVHDhigh and OVHDextra. This might be due to several reasons, such as the low number of children in our dataset living close to UNDhigh and UNDextra networks (eight out of 884) or a network misclassification due to geolocalization uncertainty (as discussed a few lines above). To definitely quantify the uncertainty error and its impact we would need the real GPS position of the child home with respect to the electric grid.

Other aspects that might have had a possible impact on the clustering described in the present study are the criteria used to classify the different types of networks, in particular the cut-off levels on the distance between child home and the electric networks. The classification criteria adopted in the present study were those already used in the EXPERS study from which the current dataset comes from. The rationale behind the choice of the classification criteria is explained in details in the original papers [5] and [20] of the EXPERS study. In particular in [5] it is explained that the “distance is defined by RTE in such a way that MF generated by these electrical networks could be negligible (below 0.1 µT).” In [20] these cut-off distances are defined as zone limit for the ‘calculated residential exposure’ criterion (m): it is explained that the distances were determined with a maximalist hypothesis of line geometries and current. In the present study, we made the decision to perform the clustering by using the same classes of electric networks already defined in the past EXPERS study because we aimed to see how the clustering approach might provide deeper insights in the results described in the past EXPERS study. In a further study with a new experimental setup and new measurements, it would be interesting to go deeper in tuning the clustering variables, for example by assessing how a different network classification (e.g., by using different cut-off values on the distances between the electric lines and the child and/or using different cut-off values of the voltages) would impact the clusters.

Last but not least, we would like to draw some comments on the sensitivity of the exposimeter and how the sensitivity might have an impact on the composition of the clusters. For what concerns the broadband and 50 Hz components (see Table 2 and Table 3), the measurements in Cluster 1 (highest exposure levels) and Cluster 2 (mid exposure levels) had mean, Q1, and Q3 values of GM above the lower threshold of sensitivity. Measurements assigned to Cluster 3 (lowest exposure levels) had mean and Q3 values above the threshold for sensitivity whereas Q1 was below the sensitivity threshold. Therefore, combining all this evidence we can say that the clusters generated from the 50 Hz and broadband components of the MF really grouped together measurements fairly well above the sensitivity threshold of the exposimeter and should have been less influenced by the measurement device. Differently, the clusters generated by the harmonic components (see Table 4) seem to be less important because the mean, Q1 and for some clusters even the Q3 values of GM were below the sensitivity threshold. This is in line with what we observed from the analysis of the correlation ratio which showed that the harmonic component was not relevant alone in differentiating the clusters.

## 5. Conclusions

In the present study we wanted to answer to a number of open questions that we did not address in our past preliminary study [19], that is to discover the possible contribution of ‘secondary’ environmental variables (such, indoor sources of ELF MF, physical parameters of the residences, and the family size) and broadband and harmonic components of the MF in differentiating the patterns of indoor exposure identified through cluster analysis. The main results of the present study can be summarized as follows: (1) indoor personal exposure was mainly characterized by the type of electric networks close to the child home, whereas secondary environmental variables had an effect but of a lesser extent; (2) the level of indoor exposure mainly depended on the type of the electric networks in the vicinity, being the highest exposures associated to children living close to overhead lines of high (63–150 kV), extra-high (225 kV) and ultra-high voltage (400 kV), followed by mid exposure levels associated to children living close to underground networks of low (400 V) and mid voltage (20 kV) and substations (20 kV/400 V) and the lowest exposures being associated to children living more distant from electric networks; (3) the harmonic component of the MF was not relevant alone in discriminating indoor exposure patterns; (4) although their effect was smaller than that of electric networks, home heating, residence type and family size were found to characterize indoor exposures, whereas the age of the residence was not determinant in differentiating the exposure scenarios; (5) children living in residences with electric heating, or in big flats or in larger families had generally a higher level of personal indoor exposure. As to point n. (3), it was not possible with the present data to definitely discriminate whether the low impact of the harmonic component was due to the sensitivity of the measurement device or was intrinsically due to the characteristics of the harmonic component itself. This seem to suggest the importance of using devices with higher sensitivity in future exposure campaigns to try to answer to this point. Evidences n. (4) and n. (5) seem to suggest that the measurement of environmental variables such as heating, residence type, family size should be done also in future campaigns of ELF MF exposure to gain better insights on their impact on personal exposure. For what concerns the impact of the uncertainty in the geolocalization of the electric lines, the present analysis was able to provide qualitative figures. It seems to be interesting to measure in future campaigns the real GPS position of the child with respect to the electric grid so that to exactly quantify the impact of geolocalization on the exposure patterns. Finally, it would be also interesting to go deeper in tuning the clustering variables, for example by assessing how a different classification of the electric networks would impact the pattern of exposure. As the last remark, we did not take into account other sources of indoor magnetic field such as train lines electrified at 50 Hz close to home, wiring in the buildings, or electric appliances in details. These additional variables could be of some interest to be analyzed in future studies to understand better their contribution to the indoor exposure compared to that of electric networks. For what concerns

## Figures and Tables

**Table 1:** Distribution of electric networks in the analyzed dataset. The table displays the number (and the percentage over the whole analyzed dataset of 884 children) of children that live close to each type of electric network and the maximum and median number of electric networks near the child home.

Type of Electric Network	N of Children (%) ^a^	Max Number of Networks	Median Number of Networks
UNDlow	640 (72.4)	59	3
UNDmid	406 (45.9)	27	0
UNDhigh	2 (0.2)	1	0
UNDextra	6 (0.7)	2	0
OVHDlow	444 (50.2)	16	1
OVHDmid	40 (4.5)	3	0
OVHDhigh	6 (0.7)	1	0
OVHDextra	6 (0.7)	1	0
OVHDultra	2 (0.2)	3	0
Substation	125 (14.1)	2	0

^a^ Please note that the same child might live close to more than one type of electric network; this is the reason why the sum of the numbers reported in the second column of this table is greater than the total number of analyzed children.

**Table 2:** Cluster model for the 50 Hz magnetic field exposure. The table displays the coordinates of the centroids for each of the 11 analyzed variables (from ‘UNDlow’ to ‘GM’) of the three clusters obtained from the analysis of the 50 Hz MF. Centroid coordinates are the average number of electric networks near the child home (for variables from ‘UNDlow’ to ‘Substation’) and the average GM in μT within each cluster. The rightmost column is the correlation ratio.

Variable	Centroid Coordinate (Average Value)			*η* ^2^
	Cluster 1 (N = 12) ^a^	Cluster 2 (N = 141)	Cluster 3 (N = 731)	
UNDlow	1.6	13.9	3.0	0.37
UNDmid	0.3	5.8	0.7	0.36
UNDhigh	0.0	0.0	0.0	0.00
UNDextra	0.0	0.0	0.0	0.02
OVHDlow	1.6	1.0	1.4	0.01
OVHDmid	0.2	0.0	0.1	0.00
OVHDhigh	0.5	0.0	0.0	0.50
OVHDextra	0.5	0.0	0.0	0.50
OVDHultra	0.4	0.0	0.0	0.16
Substation	0.2	0.9	0.0	0.72
GM (µT) ^b^	0.126	0.036	0.025	0.03
Q1	0.045	0.010	0 ^c^	
Q3	0.225	0.050	0.020	

^a^ Cluster size. ^b^ 50 Hz component of MF (average value, 1st and 3rd quartiles within each cluster). ^c^ The value is below the lower sensitivity threshold of the exposimeter.

**Table 3:** Cluster model for the broadband magnetic field exposure. For details, see Table 2.

Variable	Centroid Coordinate			*η* ^2^
	Cluster 1 (N = 12)	Cluster 2 (N = 141)	Cluster 3 (N = 731)	
UNDlow	1.6	13.9	3.0	0.37
UNDmid	0.2	5.8	0.7	0.36
UNDhigh	0.0	0.0	0.0	0.00
UNDextra	0.0	0.0	0.0	0.02
OVHDlow	1.6	1.0	1.4	0.01
OVHDmid	0.2	0.0	0.1	0.00
OVHDhigh	0.5	0.0	0.0	0.50
OVHDextra	0.5	0.0	0.0	0.50
OVDHultra	0.4	0.0	0.0	0.16
Substation	0.2	0.9	0.0	0.72
GM (µT) ^a^	0.129	0.041	0.028	0.03
Q1	0.048	0.010	0 ^b^	
Q3	0.233	0.060	0.030	

^a^ Broadband component of MF (average value, 1st and 3rd quartiles within each cluster). ^b^ The value is below the lower sensitivity threshold of the exposimeter.

**Table 4:** Cluster model for the harmonic component of magnetic field exposure. For details, see Table 2.

Variable	Centroid Coordinate			*η* ^2^
	Cluster 1 (N = 7)	Cluster 2 (N = 139)	Cluster 3 (N = 738)	
UNDlow	2.4	14.0	2.9	0.37
UNDmid	0.4	5.8	0.7	0.37
UNDhigh	0.0	0.0	0.0	0.00
UNDextra	0.0	0.0	0.0	0.02
OVHDlow	1.4	1.0	1.4	0.01
OVHDmid	0.0	0.0	0.1	0.01
OVHDhigh	0.1	0.0	0.0	0.02
OVHDextra	0.9	0.0	0.0	0.86
OVDHultra	0.7	0.0	0.0	0.27
Substation	0.1	0.9	0.0	0.70
GM (µT) ^a^	0.010	0.007	0.004	0.01
Q1	0 ^b^	0 ^b^	0 ^b^	
Q3	0.020	0.010	0 ^b^	

^a^ Harmonic component of MF (average value, 1st and 3rd quartiles within each cluster). ^b^ The value is below the lower sensitivity threshold of the exposimeter.

**Table 5:** Results of the association test between heating, residence type, and residence age and cluster composition.

Metric	Heating	Residence Type	Residence Age
*χ* ^2^	17.58 (*p* = 0.002)	138.49 (*p* = 2.09 × 10^−27^)	4.83 (*p* = 0.57)
*w* [0, 1.41]	0.14	0.40	0.08
*V* [0, 1]	0.10	0.28	0.05

**Table 6:** Joint percentage distribution between heating type and clusters.

Heating Type	Cluster 1	Cluster 2	Cluster 3	Heating Marginal
Non-electric	0.69 {6} (0.85) ^a^ {7}	9.94 {86} (9.37) {81}	50.75 {439} (51.17) {443}	61.38 {531}
Mixed	0.23 {2} (0.24) {2}	0.92 {8} (2.66) {23}	16.30 {141} (14.55) {126}	17.45 {151}
Electric	0.47 {4} (0.30) {3}	4.40 {38} (3.23) {28}	16.30 {141} (17.63) {152}	21.17 {183}
Cluster marginal	1.39 {12}	15.26 {132}	83.35 {721}	100.00 ^b^ {865}

^a^ Numbers under () brackets are the expected percentages and are calculated by multiplying the heating marginal by the corresponding cluster marginal; the corresponding number of children is displayed under {} brackets. ^b^ Percentages are calculated on 865 cases instead of 884 because for 19 cases we had no information on heating type (we only know that it is collective heating).

**Table 7:** Joint percentage distribution between residence type and clusters.

Residence Type	Cluster 1	Cluster 2	Cluster 3	Residence Marginal
Individual	1.13 {10} (0.68) ^a^ {6}	3.28 {29} (8.01) {71}	45.81 {405} (41.53) {367}	50.22 {444}
Terraced	0.12 {1} (0.26) {2}	1.58 {14} (3.07) {27}	17.53 {155} (15.90) {141}	19.23 {170}
Apartment in small building	0 {0} (0.14) {1}	2.49 {22} (1.62) {14}	7.69 {68} (8.42) {75}	10.18 {90}
Apartment in big building	0.11 {1} (0.28) {3}	8.60 {76} (3.25) {29}	11.66 {103} (16.84) {148}	20.37 {180}
Cluster marginal	1.36 {12}	15.95 {141}	82.69 {731}	100.00 ^b^ {884}

^a^ Numbers under () brackets are the expected percentages; the corresponding number of children is displayed under {} brackets. ^b^ Percentages are calculated on 884 cases.
